# The Use of Pessaries*Abstract of a short paper read at a meeting of the Austin District Medical Society. Dr. Wooten made most of his remarks extemporaneously, supplemental to his paper, as he went along; and delivered such an interesting lecture that, by request of the Society, he promised to reduce it to writing for publication; and we promised it for this issue, but the doctor has been so occupied with his various duties that he has not had time to fulfill his promise. We give an outline only of what was in his paper, omitting, from necessity, his very interesting off-hand lecture. We give also the discussion as taken down stenographically by Dr. Seth M. Morris, Professor of Chemistry Texas Medical College.—Ed.

**Published:** 1891-09

**Authors:** T. D. Wooten


For Daniel's Texas Medical Journal.

### PESSARIES.*

BY T. D. WOOTEN, M. D.

THE USE of pessaries, or, rather, I might say, the proper use of pessaries, was assigned me for a paper at the last meeting of this Society, and it was my wish and intention to comply with the request, but I have been so much occupied with other matters that it has been impossible for me to give the time necessary to prepare a paper worthy of the occasion. At odd moments I have jotted down a few thoughts in connection with the subject, and present them here in a crude form.

*Abstract of a short paper read at a meeting of the Austin District Medical Society. Dr. Wooten made most of his remarks extemporaneously, supplemental to his paper, as he went along; and delivered such an interesting lecture that, by request of the Society, he promised to reduce it to writing for publication; and we promised it for this issue, but the doctor has been so occupied with his various duties that he has not had time to fulfill his promise. We give an outline only of what was in his paper, omitting, from necessity, his very interesting off-hand lecture. We give also the discussion as taken down stenographically by Dr. Seth M. Morris, Professor of Chemistry Texas Medical College.—ED.

I am apprised of the great variety and conflicting opinions existing among professional men as to the use of pessaries. In the few remarks I shall make in order to bring the subject before the Society, I will refer mainly to the displacements known as retroversion and retroflection. In the first, the fundus of the uterus is thrown back upon the rectum, and retains its normal shape; retroflexion is dislocation backwards also, but is attended with flexion, curved or bent, posteriorly. Cases of this kind are very common; almost every fourth case the gynecologist is called upon to treat is in some way allied to this condition. In the displacements, the ovaries are often brought down behind and are impinged upon by the uterus, to the great suffering of the patient. The normal action of the bowels is interfered with; constipation, flatulence, etc., with their evil consequences, are often the result of this condition of the womb.

The dislocation may be attended with hyperplasia, ulceration, etc., of the uterus; there may be extensive adhesions, and the uterus be more or less fixed in this abnormal position. The os, or neck of the uterus, may be drawn up and forward against the bladder, impinging upon it in such a manner as to interfere with its functions, causing frequent and painful micturation, not infrequently resulting in chronic cystitis. With all these attendant effects of such malposition, or even but some of them, you have truly an invalid,—a suffering woman with all the protean symptoms and complex manifestations we call hysteria; and most likely the patient has a family of children dependent upon her for attention and nursing, necessitating her being more or less upon her feet, being thus far removed from the "luxuries of invalidism." Such cases are not infrequent; in fact you will find one or more, in Texas, in every little neighborhood circle, and they, with their dependent little ones, make a tender plea to our humanity and professional skill. What can we best do for their relief, is the question that concerns us.

In the above class of cases, after proper treatment in overcoming the conditions rendering the use of pessaries impracticable,—the use of pessaries properly adjusted and fitted to the necessities of each individual case, becomes almost indispensable to relief, and to the accomplishment of a final cure of the displacement.

Before the introduction or use of a pessary, the contra-indications to their use must be removed; such as inflammation involving almost any of the pelvic organs. Hyperplasia, granulations or ulcerations of the os, endometritis, adhesions, all must be gotten rid of, and the womb movable into position. Then a properly fitted pessary is a *sine qua non* to keep the uterus in position in order to improve the uterine circulation, and to give an opportunity for the uterine ligaments to shorten, and readjust themselves to the normal condition.

